# MiR-21, miR-34a, miR-198 and miR-217 as diagnostic and prognostic biomarkers for chronic pancreatitis and pancreatic ductal adenocarcinoma

**DOI:** 10.1186/s13000-015-0272-6

**Published:** 2015-04-24

**Authors:** Petra Vychytilova-Faltejskova, Igor Kiss, Sona Klusova, Jan Hlavsa, Vladimir Prochazka, Zdenek Kala, Jan Mazanec, Jitka Hausnerova, Leos Kren, Marketa Hermanova, Jiri Lenz, Petr Karasek, Rostislav Vyzula, Ondrej Slaby

**Affiliations:** Molecular Oncology II – Solid Cancers, Molecular Medicine, Central European Institute of Technology, Masaryk University, Brno, Czech Republic; Department of Comprehensive Cancer Care, Masaryk Memorial Cancer Institute, Zluty kopec 7, Brno, Czech Republic; Department of Surgery, Institutions shared with the Faculty Hospital Brno, Brno, Czech Republic; Department of Pathology, Institutions shared with the Faculty Hospital Brno, Brno, Czech Republic; First Department of Pathological Anatomy, Institutions shared with St. Anne’s Faculty Hospital, Brno, Czech Republic

**Keywords:** Pancreatic ductal adenocarcinoma, MicroRNAs, Diagnostic biomarkers, Prognostic biomarkers

## Abstract

**Background:**

Pancreatic ductal adenocarcinoma is an aggressive malignancy with late presentation, metastatic potential and very poor prognosis. Therefore, there is an urgent need for novel diagnostic and prognostic biomarkers. MicroRNAs are small non-coding RNAs that post-transcriptionally regulate gene expression. Altered expression of microRNAs has been reported in wide range of malignancies, including pancreatic ductal adenocarcinoma. The aim of this study was to analyze the expression of selected microRNAs in normal pancreas, chronic pancreatitis and pancreatic ductal adenocarcinoma tissues and evaluate their diagnostic and prognostic potential.

**Findings:**

Using quantitative real-time PCR, expression levels of 4 microRNAs were examined in 74 tumor tissues, 18 tissues of chronic pancreatitis and 9 adjacent normal tissues and correlated with clinicopathological features of patients. Expression levels of miR-21, miR-34a and miR-198 were significantly higher, whereas levels of miR-217 were significantly lower in pancreatic ductal adenocarcinomas compared to healthy tissues and tissues of chronic pancreatitis. Moreover, increased expression of miR-21 and miR-198 was significantly associated with shorter disease free survival and overall survival.

**Conclusions:**

Our data suggest that altered expression of examined microRNAs is related to neoplastic transformation and progression of the disease and these microRNAs could serve as diagnostic and prognostic biomarkers for pancreatic ductal adenocarcinoma.

**Virtual slides:**

The virtual slide(s) for this article can be found here: http://www.diagnosticpathology.diagnomx.eu/vs/1373952531543898

## Findings

### Background

Pancreatic cancer (PaC) accounts for 2.2% of all cancers and is the fourth most common cause of cancer related deaths [1]. Because of the lack of early symptoms, aggressive growth and early dissemination, most patients are diagnosed at late stages with advanced distant metastases, which make their disease surgically inoperable. Moreover, this type of cancer is known to be highly resistant to radiotherapy and chemotherapy and has one of the lowest five-year survival rate among solid cancers [2].

MicroRNAs (miRNAs) are small, non-coding RNAs that post-transcriptionally regulate gene expression. They can function as both, oncogenes or tumor suppressors, and play an important role in the regulation of many biological processes [3]. They can also serve as new diagnostic and prognostic biomarkers as well as promising therapeutic targets [4]. A growing number of studies have proved that miRNAs regulate the variety of processes involved in the development, progression and chemoresistance of PaC. In this study, expression of 4 miRNAs (miR-21, miR-217, miR-198 and miR-34a) that have been previously described to be deregulated in PaC [5-7] was quantified in tumor tissues of patients with pancreatic ductal adenocarcinoma (PDAC), healthy tissues and tissues of chronic pancreatitis (CP) with the aim to validate their diagnostic and prognostic potential.

## Materials and methods

### Patients and tissue samples

Specimens from 74 patients with PDAC (37 males, 37 females) and 18 patients with CP (12 males, 6 females) who had undergone resection from August 2001 through April 2012 at the Department of Surgery (Faculty Hospital Brno, Czech Republic) were used. Moreover, control pancreatic tissue samples without signs of inflammation or dysplastic changes from 9 patients were included. All subjects were of the same ethnicity (European descent). The ages of patients ranged between 30 and 79 years with a median of 60.5 years. Written informed consent was obtained from all patients and the study has been approved by the local Ethical Board.

### Extraction of miRNAs

Isolation of total RNA enriched for small RNAs was performed using formalin-fixed paraffin-embedded samples with more than 90% of cancerous, inflammatory or normal tissue. All samples were deparaffinized, treated with DNAse I, proteinase K and RNA extraction was undertaken using *mir*Vana miRNA Isolation Kit (Ambion Inc, Austin, TX, USA) according to the manufacturer's instructions. Concentration and purity of RNA were determined spectrophotometrically by measuring its optical density (A260/280 > 2.0, A260/230 > 1.8) using Nanodrop ND-1000 (Thermo Fisher Scientific, Waltham, MA, USA).

### Real-time quantification of miRNAs

Complementary DNA was synthesized from total RNA according to the TaqMan MicroRNA Assay protocol (Applied Biosystems, Foster City, CA, USA) using T100™ Thermal Cycler (Bio-Rad, Hercules, CA, USA). Real-Time PCR was performed according to the standard protocol using the TaqMan MicroRNA Assay kit and the Applied Biosystems 7500 Sequence Detection System (both Applied Biosystems, Foster City, CA, USA).

### Data normalization and statistical analysis

The threshold cycle data were calculated by SDS 2.0.1 software (Applied Biosystems, Foster City, CA, USA). All real-time PCR reactions were run in triplicates. The average expression levels of all measured miRNAs were normalized using miR-1233 (Assay No. 002768; Applied Biosystems, Foster City, CA, USA) and subsequently analyzed by the 2^-ΔCt^ method. Statistical differences between the levels of analyzed miRNAs were evaluated by non-parametric Mann-Whitney *U*-test and Kruskal-Wallis test. Survival analyses were carried out using the log-rank test and Kaplan-Meier plots approach. All calculations were performed using GraphPad Prism version 5.00 (GraphPad Software, San Diego, CA, USA). P-values of less than 0.05 were considered statistically significant.

## Results

### MiRNAs have potential to differentiate PDAC from CP and normal pancreas

**Figure 1:**
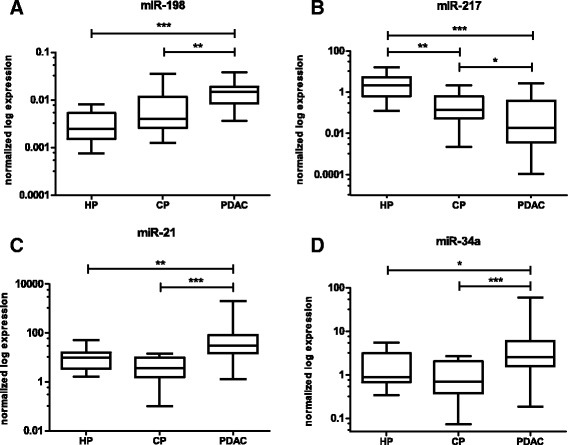
Normalized expression of analyzed miRNAs in control pancreatic tissue, CP tissue and PDAC tissue. **A)** MiR-198 is increased in PDAC compared to CP tissue (P = 0.0003; CP) and healthy pancreas (P < 0.0001; HP). **B)** MiR-217 is down-regulated in PDAC compared to CP tissue (P = 0.0307; CP) and healthy pancreas (P = 0.0001; HP). **C)** MiR-21 is up-regulated in PDAC compared to CP tissue (P < 0.0001; CP) and healthy pancreas (P = 0.0018; HP). **D)** MiR-34a is up-regulated in PDAC compared to CP tissue (P < 0.0001; CP) and healthy pancreas (P = 0.0111; HP). *P < 0.05; **P < 0.001; ***P < 0.0001.

To evaluate the diagnostic potential of 4 selected miRNAs (miR-21, miR-34a, miR-198 and miR-217), FFPE samples of 74 PDAC patients, 18 CP patients and 9 non-tumoral control pancreas were examined by qRT-PCR. Moreover, RNU48, RNU6B, RNU44, miR-1233 and miR-1260 were analyzed as potential reference genes. Using geNorm [8] and NormFinder [9], miR-1233 was selected to be the most appropriate normalization control. Using Man-Whitney *U*-test, significantly higher levels of miR-198 (P < 0.0001), miR-21 (P = 0.0018), miR-34a (P = 0.0111) and significantly lower levels of miR-217 (P = 0.0001) were observed in PDAC samples compared to healthy pancreatic tissue. Moreover, all analyzed miRNAs had the potential to differentiate PDAC from CP tissues. Whereas miR-198 (P = 0.0003), miR-21 (P < 0.0001) and miR-34a (P < 0.0001) were significantly up-regulated, miR-217 (P = 0.0307) was significantly down-regulated in PDAC samples compared to CP samples (Figure 1A-D). Subsequently, using ROC analysis, miR-21 was shown to have the highest capacity to distinguish between these two groups of samples with the sensitivity 93%, specificity 72% and AUC = 0.9227. The results of ROC analyses are summarized in Table 1.

**Table 1:** **Results of the ROC curve analysis to differentiate between pancreatic ductal adenocarcinoma and chronic pancreatitis**

**miRNA**	**AUC** ^**a**^	**Cut-off value** ^**b**^	**Sensitivity**	**Specificity**	**P-value**
miR-21	0.9227	6.3100	93.24%	72.22%	< 0.0001
miR-198	0.7748	0.0074	81.08%	72.22%	0.0003
miR-34a	0.8200	1.2400	86.67%	61.11%	< 0.0001
miR-217	0.6652	0.1205	64.86%	61.11%	0.0304

^a^AUC – area under the curve, ^b^Cut-off value – expressed as 2^-dCt^.

### MiR-21 and miR-198 can serve as new prognostic biomarkers for PDAC

**Figure 2:**
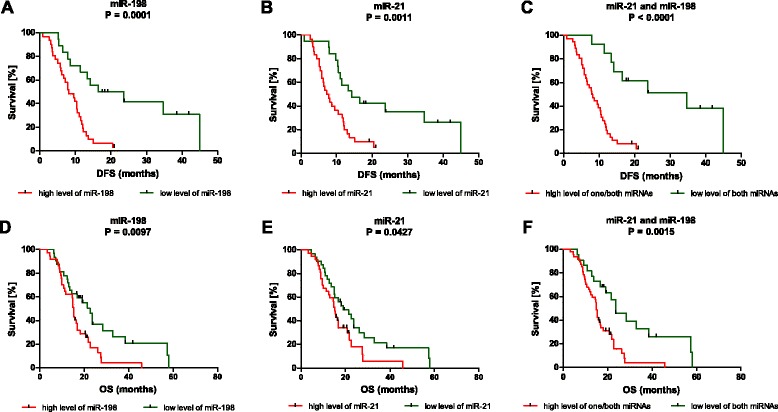
Kaplan-Meier analysis of DFS and OS based on the expression of miRNAs. **A)** PDAC patients with high level of miR-198 have shorter DFS compared to patients with low level of this miRNA (P = 0.0001). **B)** PDAC patients with high level of miR-21 have shorter DFS compared to patients with low level of this miRNA (P = 0.0011). **C)** Combined analysis of miR-21 and miR-198 – PDAC patients with low levels of both miR-21 and miR-198 have longer DFS compared to patients with high levels of miR-21 and/or miR-198 (P < 0.0001). **D)** PDAC patients with high level of miR-198 have shorter OS compared to patients with low level of this miRNA (P = 0.0097). **E)** PDAC patients with high level of miR-21 have shorter OS compared to patients with low level of this miRNA (P = 0.0427). **F)** Combined analysis of miR-21 and miR-198 – PDAC patients with low levels of both miR-21 and miR-198 have longer OS compared to patients with high levels of miR-21 and/or miR-198 (P = 0.0015).

To evaluate the prognostic function of analyzed miRNAs, Kaplan-Meier survival curves have been generated and compared by log-rank analysis. We have proven that patients with high levels of miR-21 and miR-198 have shorter both, disease free survival (DFS; P = 0.0011 for miR-21; P = 0.0001 for miR-198; Figure 2A-B) and also overall survival (OS; P = 0.0427 for miR-21; P = 0.0097 for miR-198; Figure 2D-E). Moreover, when the expression levels of these two miRNAs were combined, it has been shown that patients with low levels of both miR-21 and miR-198 have significantly higher DFS (18.2 months vs. 8 months; Figure 2C) and OS (23.7 months vs. 14.9 months; Figure 2F) compared to the patients with high levels of miR-21 and/or miR-198. Expression levels of miR-217 and miR-34a were not correlated with DFS or OS of PDAC patients (Table 2).

**Table 2:** **The detail results of Kaplan-Meier survival analysis**

**miRNA**	**2** ^**-dCt**^ **value (DFS)**	**Median DFS**	**P-value**	**2** ^**-dCt**^ **value (OS)**	**Median OS**	**P-value**
miR-21	< 22.68	14.2 months	**0.0011**	<27.15	19.1 months	**0.0427**
	>22.68	7.5 months		>27.15	15.2 months	
miR-198	<0.01208	15.3 months	**0.0001**	<0.01393	22.6 months	**0.0097**
	>0.01208	9.5 months		>0.01393	15.2 months	
miR-34a	<2.178	9.6 months	0.7186	<2.421	14.9 months	0.9320
	>2.178	11.5 months		>2.421	16.9 months	
miR-217	<0.08092	9.6 months	0.1716	<0.06097	14.9 months	0.1370
	>0.08092	12.2 months		>0.06087	21.5 months	
miR-21/miR-198	low levels of both miRNAs^a^	18.2 months	**<0.0001**	low levels of both miRNAs^b^	23.7 months	**0.0015**
	high level of one/both miRNAs^c^	8 months		high level of one/both miRNAs^d^	14.9 months	

^a^low levels of both miRNAs = level of miR-21 < 22.68 and level of miR-198 < 0.01208.

^b^low levels of both miRNAs = level of miR-21 < 27.15 and level of miR-198 < 0.01393.

^c^high level of one/both miRNAs = level of miR-21 > 22.68 or/and level of miR-198 > 0.01208.

^d^high level of one/both miRNAs = level of miR-21 > 27.15 or/and level of miR-198 > 0.01393.

## Discussion

Successful management and treatment of PDAC patients remains one of the key challenges in clinical oncology. Although the early stages of the disease can be treated surgically, most patients are diagnosed at advanced stages, when surgical resection is not possible. Moreover, differential diagnosis of pancreatic lesions is challenging [10]. Therefore, there is an urgent need for novel diagnostic biomarkers that would enable precise differential diagnosis of pancreatic lesions. In addition, molecular biomarkers that could serve as prognostic factors would be very valuable.

MiRNAs have been described to be deregulated in a variety of solid cancers, including PDAC [11-13]. In this study, the utility of miR-21, miR-34a, miR-198 and miR-217 as novel diagnostic and prognostic biomarkers of PDAC was evaluated. Consistently with the previous data, significantly increased levels of miR-21 and miR-198 [7,14,15] and decreased levels of miR-217 [7,15,16] have been observed in PDAC tissue. Despite the fact that miR-34a is generally described as an important tumor suppressor [12,17], the expression of this miRNA has been significantly higher in our PDAC samples compared to healthy tissue. Therefore, it seems that this miRNA may have dual functioning as both oncogene and tumor suppressor, depending on the cellular and tumor microenvironment [18].

Importantly, all analyzed miRNAs had a high potential to differentiate CP from PDAC tissue, therefore, they might be involved in early events of pancreatic carcinogenesis. Habbe *et al*. [19] showed that miR-21 is highly expressed in early non-invasive intraductal papillary mucinous neoplasms. Further, using *in situ* hybridization increased miR-21 expression was found in 79% of PaCs; however, only 8% of benign pancreas and 27% of CP expressed this miRNA suggesting its important role in the development of PaC [20]. MiR-217 was described to play a crucial role in regulation of acinar-to-ductal metaplasia [21], in addition, this miRNA is deregulated not only in PDAC but also in its precursor lesions, compared to non-neoplastic pancreatic tissues [15]. The function of miR-34a and miR-198 in early development of PaC has not been described till now, nevertheless, it seems that miR-21, miR-34a, miR-198 and miR-217 could be used as tumor markers to distinguish PDAC and its precursors from a benign lesions.

Given the dismal prognosis of PaC, second aim of this study was to identify miRNAs with the potential to differentiate between patients with good (DFS ≥ 12 months, OS ≥ 18 months) and poor (DFS < 12 months, OS < 18 months) prognosis. We proved that high levels of miR-21 and/or miR-198 significantly correlate with poor prognosis. Concerning miR-21, several studies have been previously published demonstrating prognostic function of this miRNA in PaC [12,20]. Moreover, high levels of miR-21 were associated with a poor response to gemcitabine and its levels were increased after the exposure to this drug [22,23]. Concerning miR-198, there are two contradictory reports analyzing the prognostic function of this miRNA in PDAC. Whereas Marin-Müller *et al*. [24] described high levels of this miRNA to be associated with good prognosis, Schultz *et al*. [7] observed correlation between over-expression of miR-198 and poor prognosis. Taken together, our data indicate that miR-21 and miR-198 could be used as potential prognostic biomarkers in PDAC patients. Importantly, the value of clinical utility of these miRNAs could be enhanced by measurement prior to resection in PDAC tissue obtained by endoscopic ultrasound-guided fine needle aspirates [25] with the aim to improve the clinical management of borderline resectable cases and identification the patients who will benefit most from the surgical resection.

## Abbreviations

PaC: Pancreatic cancer
MiRNA: MicroRNA
PDAC: Pancreatic ductal adenocarcinoma
CP: Chronic pancreatitis
DFS: Disease free survival
OS: Overall survival

## References

[1] Yadav D, Lowenfels AB (2013). The Epidemiology of Pancreatitis and Pancreatic Cancer. Gastroenterology.

[2] Siegel R, Ma J, Zou Z, Jemal A (2014). Cancer statistics, 2014. CA Cancer J Clin.

[3] Osada H, Takahashi T (2007). MicroRNAs in biological processes and carcinogenesis. Carcinogenesis.

[4] Sun T, Kong X, Du Y, Li Z (2014). Aberrant MicroRNAs in Pancreatic Cancer: Researches and Clinical Implications. Gastroenterol Res Pract.

[5] Chang T-C, Wentzel EA, Kent OA, Ramachandran K, Mullendore M, Lee KH (2007). Transactivation of miR-34a by p53 broadly influences gene expression and promotes apoptosis. Mol Cell.

[6] Hwang J-H, Voortman J, Giovannetti E, Steinberg SM, Leon LG, Kim Y-T (2010). Identification of microRNA-21 as a biomarker for chemoresistance and clinical outcome following adjuvant therapy in resectable pancreatic cancer. PloS One.

[7] Schultz NA, Werner J, Willenbrock H, Roslind A, Giese N, Horn T (2012). MicroRNA expression profiles associated with pancreatic adenocarcinoma and ampullary adenocarcinoma. Mod Pathol Off J U S Can Acad Pathol Inc.

[8] Vandesompele J, Preter KD, Pattyn F, Poppe B, Roy NV, Paepe AD, et al. Accurate normalization of real-time quantitative RT-PCR data by geometric averaging of multiple internal control genes. Genome Biol. 2002;3:research0034. 10.1186/gb-2002-3-7-research0034 PMC126239 12184808

[9] Andersen CL, Jensen JL, Ørntoft TF (2004). Normalization of Real-Time Quantitative Reverse Transcription-PCR Data: A Model-Based Variance Estimation Approach to Identify Genes Suited for Normalization, Applied to Bladder and Colon Cancer Data Sets. Cancer Res.

[10] Van Gulik TM, Reeders JW, Bosma A, Moojen TM, Smits NJ, Allema JH (1997). Incidence and clinical findings of benign, inflammatory disease in patients resected for presumed pancreatic head cancer. Gastrointest Endosc.

[11] Wang J, Paris PL, Chen J, Ngo V, Yao H, Frazier ML, et al. Next generation sequencing of pancreatic cyst fluid microRNAs from low grade- benign and high grade- invasive lesions. Cancer Lett. 2015;356:404-9. 10.1016/j.canlet.2014.09.029 PMC6200344 25304377

[12] Jamieson NB, Morran DC, Morton JP, Ali A, Dickson EJ, Carter CR (2012). MicroRNA molecular profiles associated with diagnosis, clinicopathologic criteria, and overall survival in patients with resectable pancreatic ductal adenocarcinoma. Clin Cancer Res Off J Am Assoc Cancer Res.

[13] Bloomston M, Frankel WL, Petrocca F, Volinia S, Alder H, Hagan JP (2007). MicroRNA expression patterns to differentiate pancreatic adenocarcinoma from normal pancreas and chronic pancreatitis. JAMA J Am Med Assoc.

[14] Sicard F, Gayral M, Lulka H, Buscail L, Cordelier P (2013). Targeting miR-21 for the therapy of pancreatic cancer. Mol Ther J Am Soc Gene Ther.

[15] Xue Y, Abou Tayoun AN, Abo KM, Pipas JM, Gordon SR, Gardner TB (2013). MicroRNAs as diagnostic markers for pancreatic ductal adenocarcinoma and its precursor, pancreatic intraepithelial neoplasm. Cancer Genet.

[16] Zhao W-G, Yu S-N, Lu Z-H, Ma Y-H, Gu Y-M, Chen J (2010). The miR-217 microRNA functions as a potential tumor suppressor in pancreatic ductal adenocarcinoma by targeting KRAS. Carcinogenesis.

[17] Lodygin D, Tarasov V, Epanchintsev A, Berking C, Knyazeva T, Körner H (2008). Inactivation of miR-34a by aberrant CpG methylation in multiple types of cancer. Cell Cycle Georget Tex.

[18] Gebeshuber CA, Zatloukal K, Martinez J (2009). miR-29a suppresses tristetraprolin, which is a regulator of epithelial polarity and metastasis. EMBO Rep.

[19] Habbe N, Koorstra J-BM, Mendell JT, Offerhaus GJ, Ryu JK, Feldmann G (2009). MicroRNA miR-155 is a biomarker of early pancreatic neoplasia. Cancer Biol Ther.

[20] Dillhoff M, Liu J, Frankel W, Croce C, Bloomston M (2008). MicroRNA-21 is overexpressed in pancreatic cancer and a potential predictor of survival. J Gastrointest Surg Off J Soc Surg Aliment Tract.

[21] Azevedo-Pouly ACP. Biological functions of microRNA-216 and microRNA-217 during the development of pancreatic cancer. Columbus, Ohio, USA: The Ohio State University; 2013.

[22] Giovannetti E, Funel N, Peters GJ, Chiaro MD, Erozenci LA, Vasile E (2010). MicroRNA-21 in Pancreatic Cancer: Correlation with Clinical Outcome and Pharmacologic Aspects Underlying Its Role in the Modulation of Gemcitabine Activity. Cancer Res.

[23] Wang P, Zhuang L, Zhang J, Fan J, Luo J, Chen H (2013). The serum miR-21 level serves as a predictor for the chemosensitivity of advanced pancreatic cancer, and miR-21 expression confers chemoresistance by targeting FasL. Mol Oncol.

[24] Marin-Muller C, Li D, Bharadwaj U, Li M, Chen C, Hodges SE (2013). A tumorigenic factor interactome connected through tumor suppressor microRNA-198 in human pancreatic cancer. Clin Cancer Res Off J Am Assoc Cancer Res.

[25] Szafranska AE, Doleshal M, Edmunds HS, Gordon S, Luttges J, Munding JB (2008). Analysis of microRNAs in pancreatic fine-needle aspirates can classify benign and malignant tissues. Clin Chem.

